# Pharmacokinetics of inhaled colistin in critically ill patients with ventilator-associated tracheobronchitis

**DOI:** 10.1186/cc10677

**Published:** 2012-03-20

**Authors:** Z Athanassa, M Fousteri, S Markantonis, P Myrianthefs, E Boutzouka, E Tsigou, A Tsakris, G Baltopoulos

**Affiliations:** 1 Hygeia Hospital, Marousi, Greece; 2 Faculty of Pharmacy, University of Athens, Greece; 3 Faculty of Nursing, University of Athens, Greece; 4 Faculty of Medicine, University of Athens, Greece

## Introduction

Although inhaled colistin is frequently used in ventilator-associated pneumonia (VAP), data regarding its pharmacokinetic properties are scarce [1-3]. The aim of this study was to describe colistin pharmacokinetics in critically ill patients after administration of a single dose of 1 million units of colistimethate sodium (CMS) via nebulization.

## Methods

Patients with ventilator-associated tracheobronchitis dye to polymyxin-only susceptible Gram-negative bacteria were included in the study; patients receiving intravenous and/or nebulized colistin were excluded. CMS was administered at a dose of 1 million units every 8 hours for 7 days, via a vibrating-mesh nebulizer. Mini bronchoalveolar lavage was collected before and at 1, 4 and 8 hours post nebulization, while blood samples were collected before and at 0.16, 0.5, 1, 2, 4, and 8 hours post nebulization. Colistin concentrations in epithelial lining fluid (ELF) and plasma were determined by high-performance liquid chromatography.

## Results

Our study population included five patients (three female) with mean age 60.6 years. Median (range) colistin concentrations in ELF were 6.9 (6.2 to 13.9), 3.7 (2.7 to 11.6) and 2.1 (1.2 to 8.7) g/ml at 1, 4, and 8 hours, respectively, after nebulization. Colistin concentrations in serum were substantially lower than those observed in ELF with peak median (range) values 1.56 (1.19 to 2) g/ml. The estimated colistin mean half-life was 3.4 hours.

## Conclusion

Administration of 1 million units of inhaled CMS resulted in high colistin concentrations in the ELF; moreover, concentrations were maintained for up to 8 hours in the majority of patients. This finding might support the use of inhaled CMS for the treatment of patients with VAP due to multidrug-resistant Gram-negative bacteria. Moreover, the low serum concentrations and the short half-life suggest that administration of inhaled colistin may be associated with less systemic toxicity.
